# The psychological implications of COVID-19 over the eighteen-month time span following the virus breakout in Italy

**DOI:** 10.3389/fpsyg.2024.1363922

**Published:** 2024-05-07

**Authors:** Ingrid Ropi, Margherita Lillo, Matteo Malavasi, Alessandro Argentieri, Aurora Barbieri, Baowen Lou, Diego Maria Barbieri, Marco Passavanti

**Affiliations:** ^1^Illedi HNP Srl, Giulianova, Italy; ^2^Department of Psychology, Universität Greifswald, Greifswald, Germany; ^3^Associazione Nazionale Professionale di Antropologia (ANPIA), Bologna, Italy; ^4^Department of Agricultural Economic, Agrarian University of Ecuador, Guayaquil, Ecuador; ^5^Faculty of Medicine and Surgery, University of Modena and Reggio Emilia, Modena, Italy; ^6^Department of Civil and Environmental Engineering, Norwegian University of Science and Technology, Trondheim, Norway; ^7^Department of Built Environment, Oslo Metropolitan University OsloMet, Oslo, Norway; ^8^Centro Armonico Terapeutico (CAT), Campogalliano, Italy

**Keywords:** COVID-19, depression, stress, anxiety, psychological impact

## Abstract

**Background:**

In a short time, the COVID-19 pandemic has exerted a huge impact on many aspects of people’s lives with a number of consequences, an increase in the risks of psychological diseases being one of them. The aim of this experimental study, based on an eighteen-month follow-up survey, is to assess the psychological effects of the COVID-19 pandemic, in particular, changes in stress, anxiety and depression levels, and the risks of developing Post-Traumatic Stress Disorder (PTSD).

**Methods:**

A follow-up survey was performed on a sample of 184 Italian individuals to collect relevant information about the psychological impact of COVID-19. Predictors of the components of the psychological impact were calculated based on the ANCOVA model.

**Results:**

The analysis of the online questionnaires led to the conclusion that a high percentage of the participants suffer from levels of stress, anxiety and depression higher than normal as well as an increased risk of PTSD. The severity of such disorders significantly depends on gender, the loss of family members or acquaintances due to the pandemic, the amount of time spent searching for COVID-19 related information, the type of information sources and, in part, on the level of education and income. The time factor had a more severe effect on the low-income population.

**Conclusion:**

COVID-19 has entailed a very strong psychological impact on the Italian population also depending on the coping strategies adopted, the level of mindful awareness, socio-demographic variables, people’s habits and the way individuals use the available means of communication and information.

## 1 Introduction

Coronavirus Disease 2019 (COVID-19) was first identified in December 2019. In January 2020, a new type of virus from the coronavirus family, SARS-CoV-2, was clearly identified (Huang et al., 2020). After the initial spreading, COVID-19 quickly evolved into a global pandemic, with the World Health Organization (WHO) declaring it a global health emergency on January 30, 2020 (Sohrabi et al., 2020).

As of December 27, 2021, there were over 276 million confirmed cases and more than 5 million reported deaths from COVID-19 worldwide and there were approximately 6 million confirmed cases and nearly 140,000 deaths in Italy (WHO, 2021). The spread of COVID-19 was facilitated by contemporary travel and transportatsystems (Bielecki et al., 2021; Sharun et al., 2021).

Government responses included lockdowns, with Italy experiencing its first outbreak in Lombardy region in February 2020 (Romagnani et al., 2020).

The first period of total lockdown, the following partial reopening as well as the changing regulations enacted by different government policies regarding containment measures necessarily had important implications and impacts on people’s psyche (Xiong et al., 2020; Passavanti et al., 2021). Such effects were predominantly negative, increasing problems related to a wide range of mental disorders such as generalized stress, anxiety, depression, Post-Traumatic Stress Disorder (PTSD), insomnia, and increased suicidal tendencies in people already suffering from psychological issues (Tee et al., 2020; Xiong et al., 2020).

Isolation and quarantine measures, which are commonly implemented during epidemics, generate separation and restriction of movement between human beings imposing, as a consequence, drastic changes in daily routines and requiring a psychic adaptation to the new living condition in a context of great physical, social, economic and psychological vulnerability (Passavanti et al., 2021; Gonçalves et al., 2022).

During the COVID-19 epidemic, scientists around the world performed surveys and questionnaires to understand the effects of the pandemic to provide useful answers and critical insights as quickly as possible to the population and governments (Pakenham et al., 2020; Akintunde et al., 2021; Chen et al., 2021).

Analyses of many studies have also revealed a tight correlation between the use of social networks, and digital tools in general, and the increase or decrease in people’s psychological distress, especially during periods of total lockdown (Planchuelo-Gómez et al., 2020; Tee et al., 2020; Himelein-Wachowiak et al., 2021; Passavanti et al., 2021). Furthermore, a clear correlation was found between the increase or decrease in disorders based on the type of use of social platforms used to obtain information and news about the ongoing pandemic and the evolution of the situation (Chand, 2021; Passavanti et al., 2021).

In accordance with the scientific literature reviewed, there are other factors that weigh on people’s mental health: the economic consequences on household incomes, the increasing uncertainty about pandemic development especially among young people, the greater psychological impact in some social categories such as doctors, nurses, university students, pregnant women, unemployed individuals (Cao et al., 2020; Xiong et al., 2020; Dean et al., 2021).

Previous studies suggest that lower levels of social and mental support, coupled with a heightened perception of risk, tend to correlate with the development of psychological symptoms (Bentenuto et al., 2021).

Moreover, research in the scientific literature suggests that various factors could contribute to psychological symptoms. These include coping mechanisms (Ho et al., 2020), individual temperament and attachment style (Moccia et al., 2020), insufficient information or rumours circulated on social media (Brooks et al., 2020; Roy et al., 2020), awareness and mindfulness abilities (Moccia et al., 2020; Wang C. et al., 2020; Wang H. et al., 2020; Passavanti et al., 2021).

Finally, lockdowns implemented to control the spread of coronavirus disease 2019 (COVID-19) have had profound effects on daily life worldwide. However, their impact on mental health remains unclear as available meta-analyses and reviews are primarily based on cross-sectional studies, despite some attempts to analyse its longitudinal effects (Prati and Mancini, 2021; Reagu et al., 2021).

This longitudinal study on an Italian sample concerns a second phase of a previously published international research effort in 2020 and enables to understand the evolution of the pandemic from both a social and a psychological perspective over an eighteen-month time span following the COVID-19 breakout in Italy (Passavanti et al., 2021).

Therefore, this study aims to investigate how variables may be impactful over a longer period: sociodemographic differences (gender, education, income), personality traits (coping strategies and mindfulness), situational factors (experiencing contagion or loss of family members), behavioral aspects (internet and social media usage, access to information).

By integrating these hypotheses into the study design, it is possible to obtain richer and contextualized data on the psychological impact of the pandemic, allowing for a better understanding of the factors influencing the mental health and well-being of those involved.

## 2 Methods

### 2.1 Setting, participants and procedure

The study was conducted by means of an online follow-up survey to Italian respondents who had participated in the first phase of this research performed in the spring of 2020 (Passavanti et al., 2021).

The follow-up survey questionnaire involved an exclusively Italian sample thus differing from the previous research which also included participants from six other countries. Google Forms was the online platform chosen for the administration of the questionnaires. Out of the initial 420 Italian survey population (Passavanti et al., 2021), a sample of 184 responded to the second survey between December 15, 2021 and December 30, 2021.

The personal data were collected, aggregated, and analysed anonymously, in observance of the ethical principles of the Declaration of Helsinki for medical research on human subjects.

Only adult participants were allowed to join the survey, and informed written consent was obtained from each of them. Participants were given the freedom to choose whether or not to participate in the questionnaire posed minimal risk and could withdraw at any time.

The survey study underwent review and was approved by a major institutional board, namely the Norwegian Centre for Research Data.

### 2.2 Variables and metrics

Administered to participants from various Italian regions, the questionnaire explored different psychological aspects and consisted of three main sections:

1. **Socio-demographic information:** this section collected data on participants’ gender, nationality, age, education, knowledge of infected individuals, and their relationship with technology.
2. **Relationship with social networks and leisure time:** this part delved into participants’ engagement with social networks and their leisure activities.
3. **Specific use of media and technologies during the pandemic period:** the final section focused on participants’ utilization of media and technology during the pandemic, shedding light on their relationship with digital tools, especially during quarantine and lockdowns.

The survey was entirely written and administered in Italian. Anyway, the questions are translated into English in Tables 1, 2.

**Table 1:** Association between social demographics characteristics and the psychological impact of pandemic on the PSS10, PHQ-9 and IES-R.

	PSS10					PHQ-9					IES-R				
Variables	*F*	*p*	*M*	SE	95%CI	*F*	*p*	*M*	SE	95%CI	*F*	*p*	*M*	SE	95%CI
**Gender**	16.017	<0.001 ***				13.434	<0.001 ***				7.002	0.009 **			
Male			19.815	0.730	18.380 to 21.250			8.674	0.533	7.626 to 9.722			28.561	1.952	24.723 to 32.400
Female			23.258	0.546	22.183 to 24.333			10.896	0.388	10.133 to 11.660			34.537	1.480	31.626 to 37.448
**Education**	1.220	0.297				4.579	0.011*				0.874	0.418			
<=High school			21.448	0.761	19.950 to 22.945			9.764	0.554	8.673 to 10.854			31.336	2.026	27.350 to 35.322
Bachelor’s degree			22.257	0.710	20.859 to 23.654			10.823	0.515	9.811 to 11.835			33.240	1.840	29.622 to 36.858
> = Master’s degree			20.905	0.635	19.656 to 22.154			8.769	0.512	7.761 to 9.776			30.072	1.893	26.348 to 33.796
**Declared income**	0.386	0.680				1.730	0.179				0.763	0.467			
Low			21.732	0.583	20.585 to 22.878			10.390	0.457	9.490 to 11.289			30.435	1.719	27.055 to 33.816
Medium			21.911	0.629	20.674 to 23.148			9.288	0.456	8.391 to 10.184			30.444	1.449	27.593 to 33.295
High			20.966	0.973	19.052 to 22.881			9.678	0.693	8.314 to 11.042			33.768	2.685	28.486 to 39.051
**Are you acquainted with a person who died because of COVID-19?**	4.712	0.031*				4.676	0.031*				2.698	0.101			
No			22.307	0.593	21.140 to 23.473			10.365	0.486	9.410 to 11.321			33.128	1.618	29.945 to 36.311
Yes			20.766	0.601	19.585 to 21.948			9.205	0.398	8.422 to 9.988			29.971	1.636	26.753 to 33.189
**How long did you use smartphone and computer to keep in touch and/or stay on social networks since the epidemic restrictions started?**	1.426	0.235				4.297	0.005 **				0.809	0.489			
Less than one hour per day			19.529	1.276	17,019 to 22.040			7.126	0.954	5.251 to 9.002			31.214	3.190	24.941 to 37.488
Between one and two hours per day			22.360	0.780	20.826 to 23.894			10.485	0.501	9.499 to 11.471			30.696	2.330	26.114 to 35.279
Between two and five hours			21.987	0.683	20.643 to23.330			10.711	0.462	9.801 to 11.620			30.394	1.505	27.433 to 33.355
More than five hours			22.270	0.674	20.945 to 23.595			10.819	0.592	9.655 to 11.983			33.893	2.060	29.842 to 37.944
**How often do you search for information about the progress of the epidemic?**	0.900	0.407				2.884	0.057				9.623	<0.001 ***			
Rarely (less than 3 times a week)			20.832	0.732	19.392 to 22.272			8.801	0.529	7.761 to 9.841			25.707	1.926	21.918 to 29.496
Once a day			21.945	0.665	20.636 to 23.254			10.180	0.515	9.167 to 11.194			32.646	2.048	28.617 to 36.674
Many times a day			21.833	0.759	20.339 to 23.326			10.374	0.558	9.276 to 11.472			36.295	1.836	32.683 to 39.907
**Time**	1.909	0.168				0.008	0.928				12.255	0.001 **			
Time 1 (Apr. 2020)			20.975	0.670	19.658 to 22.293			9.805	0.458	8.904 to 10.706			34.262	1.726	30.866 to 37.658
Time 2 (Dec. 2021)			22.098	0.585	20.947 to 23.249			9.765	0.376	9.025 to 10.505			28.836	1.292	26.295 to 31.378
Gender*Time	0.982	0.322				0.212	0.645				0.724	0.395			
Male*Time1			18.920	0.968	17.016 to 20.823			8.799	0.719	7.386 to 10.213			30.648	2.593	25.549 to 35.748
Male*Time2			20.710	0.835	19.068 to 22.353			8.548	0.555	7.457 to 9.640			26.474	1.933	22.672 to 30.276
Female*Time1			23.031	0.815	21.428 to 24.633			10.812	0.496	9.835 to 11.788			37.876	1.913	34.114 to 41.639
Female*Time2			23.485	0.692	22.124 to 24.846			10.981	0.457	10.083 to 11.880			31.199	1.564	28.123 to 34.275
Education*Time	0.766	0.466				0.572	0.565				0.299	0.742			
<=High School*Time1			20.428	1.166	18.134 to 22.722			9.986	0.817	8.379 to 11.592			33.554	2.671	28.301 to 38.807
<=High School*Time2			22.468	0.887	20.724 to 24.212			9.541	0.566	8.429 to 10.654			29.118	1.937	25.308 to 32.929
Bachelor’s degree*Time1			22.095	0.976	20.176 to 24.014			11.035	0.650	9.756 to 12.313			36.534	2.435	31.745 to 41.324
Bachelor’s degree*Time2			22.419	0.714	21.015 to 23.086			10.612	0.558	9.515 to 11.708			29.945	1.927	26.156 to 33.735
> = Master’s degree*Time1			20.403	0.883	18.666 to 22.140			8.396	0.721	6.979 to 9.813			32.698	2.382	28.013 to 37.384
> = Master’s degree*Time2			21.407	0.854	19.728 to 23.086			9.141	0.609	7.943 to 10.340			27.446	2.025	23.462 to 31.429
Declared Income*Time	2.315	0.100				6.139	0.002 **				0.539	0.584			
Low*Time1			20.263	0.834	18.623 to 21.903			9.365	0.612	8.161 to 10.570			32.239	2.179	27.952 to 36.525
Low*Time2			23.201	0.695	21.834 to 23.568			11.414	0.511	10.410 to 12.419			28.632	1.844	25.006 to 32.258
Medium*Time1			21.785	0.893	20.029 to 23.541			9.618	0.623	8.393 to 10.844			33.580	1.847	29.947 to 37.213
Medium*Time2			22.037	0.719	20.623 to 23.451			8.957	0.506	7.961 to 9.953			27.309	1.662	24.040 to 30.577
High*Time1			20.877	1.398	18.128 to 23.627			10.433	0.910	8.643 to 12.222			36.968	3.526	30.033 to 43.903
High*Time2			21.056	1.127	18.840 to 23.272			8.923	0.752	7.444 to 10.402			30.569	2.603	25.448 to 35.690
Are you acquainted with a person who died because of COVID-19?*Time	0.390	0.533				0.703	0.403				0.375	0.541			
No*Time1			21.931	0.875	20.209 to 23.653			10.569	0.654	9.283 to 11.855			36.229	2.151	31.997 to 40.461
No*Time2			22.682	0.669	21.367 to 23.997			10.162	0.475	9.228 to 11.096			30.027	1.623	26.835 to 33.219
Yes*Time1			20.019	0.805	18.435 to 21.604			9.042	0.531	7.998 to 10.086			32.296	2.080	28.204 to 36.388
Yes*Time2			21.514	0.760	20.018 to 23.009			9.368	0.497	8.390 to 10.345			27.646	1.736	24.232 to 31.060
How long did you use smartphone and PC and/or stay on social networks since the epidemic restrictions started?*Time	1.238	0.296				2.149	0.094				0.507	0.678			
Less than one hour per day*Time1			17.472	1.685	14.157 to 20.787			6.423	1.069	4.320 to 8.526			32.797	4.160	24.615 to 40.978
Less than one hour per day*Time2			21.587	1.648	18.345 to 24.829			7.829	1.036	5.792 to 9.866			29.632	3.177	23.383 to 35.880
Between one and two hours per day*Time1			22.882	1.319	20.288 to 25.477			11.413	0.808	9.824 to 13.002			35.037	3.080	28.979 to 41.095
Between one and two hours per day*Time2			21.838	1.064	19.745 to 23.931			9.556	0.605	8.365 to 10.747			26.355	2.622	
Between two and five hours*Time1			21.731	0.960	19.843 to 23.620			10.693	0.669	9.378 to 12.008			33.061	1.959	
Between two and five hours*Time2			22.242	0.616	21.030 to 23.454			10.729	0.486	9.772 to 11.686			27.727	1.687	
More than five hours*Time1			21.816	0.876	20.092 to 23.540			10.693	0.784	9.151 to 12.235			36.155	2.572	
More than five hours*Time2			22.724	0.827	21.097 to 24.351			10.944	0.670	9.626 to 12.263			31.631	2.085	
How often do you search for information about the progress of the epidemic?*Time	0.982	0.376				0.655	0.520				1.979	0.140			
Rarely *Time1			20.246	1.063	18.156 to 22.337			9.067	0.712	7.667 to 10.466			30.155	2.422	25.392 to 34.919
Rarely *Time2			21.418	0.883	19.682 to 23.154			8.534	0.601	7.353 to 9.716			21.260	2.000	17.326 to 25.193
Once a day*Time1			20.875	0.945	19.016 to 22.734			9.863	0.701	8.485 to 11.242			34.706	2.674	29.447 to 39.966
Once a day*Time2			23.014	0.797	21.447 to 24.582			10.498	0.572	9.372 to 11.623			30.585	2.076	26.502 to 34.668
Many times a day*Time1			21.804	1.033	19.773 to 23.836			10.486	0.728	9.055 to 11.918			37.926	2.474	33.059 to 42.792
Many times a day*Time2			21.861	0.832	20.225 to 23.497			10.262	0.568	9.144 to 11.380			34.664	1.936	30.857 to 38.472
MAAS	20.034	<0.001***				16.236	<0.001***				4.998	0.026*			
Brief-COPE Approach	9.199	0.003 **				0.376	0.540				1.230	0.268			
Brief-COPE Avoidant	60.446	<0.001***				50.178	<0.001***				28.071	<0.001***			

Results refer to the three regression linear models with PSS10, PHQ-9 and IES-R as dependent variables, linked to the ANCOVA model. Covariates in the model are MAAS, Brief-COPE Avoidant and Brief-COPE Approach. Covariates are evaluated based on the following values: MAAS = 57, Brief-COPE Avoidant = 27, Brief-COPE Approach = 36. *T*-tests are evaluated at 5% (*p < 0.05), 1% (**p < 0.01), and 0.1% (***p > 0.001).

**Table 2:** Association between social demographics characteristics and the psychological impact of the pandemic on the DASS-21 subscales.

		DASS-21 stress					DASS-21 depression					DASS-21 anxiety			
Variables	*F*	*p*	*M*	SE	95% CI	*F*	*p*	*M*	SE	95% CI	*F*	*p*	*M*	SE	95% CI
															
**Gender**	14.070	<0.001 ***				7.975	0.005 **				8.025	0.005 **			
Male			17.021	1.209	14.642 to 19.399			14.724	1.305	12.687 to 16.760			6.184	0.950	4.945 to 8.683
Female			22.323	0.822	20.706 to 23.939			18.301	0.906	16.518 to 20.083			10.046	0.833	8.408 to 11.684
**Education**	3.565	0.029*				2.638	0.073				1.769	0.172			
<=High School			18.501	1.182	16.177 to 20.825			16.696	1.204	14.327 to 19.065			9.332	1.166	7.039 to 11.625
Bachelor’s degree			21.815	1.088	19.675 to 23.956			18.041	1.120	15.838 to 20.244			8.830	1.012	6.840 to 10.819
> = Master’s degree			18.699	1.052	16.629 to 20.769			14.799	1.021	12.791 to 16.807			7.129	0.913	5.333 to 8.925
**Declared Income**	0.207	0.813				0.478	0.620				0.466	0.628			
Low			20.201	0.840	18.548 to 21.854			17.255	0.931	15.424 to 19.087			9.041	0.910	7.252 to 10.830
Medium			19.759	0.923	17.943 to 21.576			16.100	0.977	14.178 to 18.023			7.924	0.769	6.413 to 9.436
High			19.056	1.715	15.682 to 22.429			16.181	1.539	13.153 to 19.208			8.325	1.497	5.382 to 11.269
**Are you acquainted with a person who died because of COVID-19?**	2.022	0.156				6.996	0.009 **				0.755	0.386			
No			20.450	0.891	18.698 to 22.201			17.988	0.939	16.141 to 19.835			8.880	0.866	7.177 to 10.583
Yes			18.894	0.972	16.982 to 20.806			15.036	0.912	13.241 to 16.830			7.980	0.856	6.297 to 9.664
**How long did you use smartphone and computer to keep in touch and/or stay on social networks since the epidemic restrictions started?**	2.516	0.058				1.173	0.320				2.463	0.062			
Less than one hour per day			16.422	1.608	13.259 to 19.586			13.945	2.024	9.964 to 17.926			5.824	1.558	2.759 to 8.889
Between one and two hours per day			21.544	1.231	19.123 to 23.965			17.739	1.153	15.471 to 20.007			8.898	1.141	6.653 to 11.143
Between two and five hours			20.174	0.929	18.346 to 22.002			16.697	0.948	14.831 to 18.562			8.266	0.811	6.671 to 9.861
More than five hours			20.547	1.401	17.792 to 23.302			17.667	1.218	15.271 to 20.064			10.733	1.234	8.307 to 13.159
**How often do you search for information about the progress of the epidemic?**	5.654	0.004 **				1.981	0.140				1.172	0.311			
Rarely (Less than 3 times a week)			16.844	1.132	14.618 to 19.070			14.962	1.141	12.718 to 17.205			7.400	1.042	5.351 to 9.450
Once a day			20.366	1.171	18.062 to 22.670			17.908	1.099	15.747 to 20.069			8.288	1.069	6.186 to 10.390
Many times a day			21.806	1.168	19.507 to 24.104			16.666	1.167	14.371 to 18.962			9.602	1.104	7.432 to 11.773
**Time**	0.413	0.521				0.128	0.721				0.004	0.951			
Time 1 (Apr. 2020)			19.309	1.035	17.274 to 21.345			16.701	1.043	14.650 to 18.753			8.459	0.943	6.604 to 10.314
Time 2 (Dec. 2021)			20.034	0.839	18.384 to 21.685			16.323	0.750	14.848 to 17.798			8.401	0.699	7.027 to 9.776
Gender*Time	0.003	0.958				1.045	0.307				2.183	0.140			
Male*Time1			16.686	1.586	13.607 to 19.766			15.417	1.444	12.576 to 18.258			7.429	1.271	4.928 to 9.929
Male*Time2			17.356	1.394	14.614 to 20.098			14.030	1.107	11.853 to 16.208			6.200	1.018	4.198 to 8.201
Female*Time1			21.933	1.154	19.663 to 24.203			17.985	1.264	15.500 to 20.471			9.489	1.125	7.276 to 11.703
Female*Time2			22.713	0.951	20.843 to 24.582			18.616	0.985	16.678 to 20.553			10.603	0.876	8.879 to 12.327
Education*Time	0.340	0.712				1.316	0.270				2.621	0.074			
<=High School*Time1			17.783	1.650	14.492 to 20.984			16.586	1.667	13.306 to 19.866			9.176	1.196	6.038 to 12.315
<=High School*Time2			19.264	1.366	16.578 to 21.951			16.805	1.390	14.072 to 19.539			9.487	1.306	6.918 to 12.057
Bachelor’s degree*Time1			21.892	1.453	19.033 to 24.750			19.144	1.544	16.107 to 22.182			10.030	1.385	7.306 to 12.753
Bachelor’s degree*Time2			21.739	1.195	19.389 to 24.090			16.938	1.116	14.742 to 19.134			7.629	1.062	5.541 to 9.718
> = Master’s degree*Time1			18.299	1.449	15.450 to 21.148			14.373	1.436	11.548 to 17.198			6.171	1.172	3.866 to 8.476
> = Master’s degree*Time2			19.099	1.268	16.605 to 21.593			15.225	1.139	12.984 to 17.466			8.087	1.089	5.946 to 10.228
Declared Income*Time	1.272	0.282				3.219	0.041*				3.261	0.040*			
Low*Time1			18.851	1.146	16.598 to 21.104			15.720	1.285	13.193 to 18.247			7.715	1.117	5.518 to 9.912
Low*Time2			21.550	1.071	19.444 to 23.656			18.791	1.049	16.728 to 20.854			10.367	1.070	8.263 to 12.471
Medium*Time1			19.813	1.308	17.241 to 22.386			16.648	1.316	14.049 to 19.236			8.833	1.097	6.676 to 10.991
Medium*Time2			19.705	1.110	17.521 to 21.889			15.553	1.068	13.451 to 17.655			7.015	0.852	5.339 to 8.691
High*Time1			19.264	2.213	14.911 to 23.617			17.736	2.183	13.442 to 22.031			8.829	1.985	4.924 to 12.733
High*Time2			18.847	1.820	15.267 to 22.428			14.625	1.616	11.445 to 17.804			7.822	1.631	4.613 to 11.031
Are you acquainted with a person who died because of COVID-19?*Time	0.122	0.727				2.740	0.099				0.404	0.525			
No*Time1			20.246	1.254	17.778 to 22.713			18.896	1.329	16.282 to 21.511			9.168	1.157	6.892 to 11.445
No*Time2			20.654	1.046	18.597 to 22.711			17.080	0.957	15.197 to 18.963			8.592	0.937	6.749 to 10.434
Yes*Time1			18.373	1.301	15.814 to 20.933			14.506	1.252	12.044 to 19.968			7.749	1.175	5.438 to 10.061
Yes*Time2			19.414	1.100	17.252 to 21.577			15.566	1.025	13.549 to 17.582			8.211	0.947	6.349 to 10.073
How long did you use smartphone and computer to keep in touch and/or stay on social networks since the epidemic restrictions started?*Time	1.695	0.168				0.475	0.700				0.959	0.412			
Less than one hour per day*Time1			14.140	2.449	9.323 to 18.958			13.503	2.797	8.061 to 18.946			7.108	2.278	2.628 to 11.588
Less than one hour per day*Time2			18.705	1.786	15.193 to 22.217			14.386	1.794	10.857 to 17.916			4.539	1.221	2.138 to 6.941
Between one and two hours per day*Time1			22.356	1.782	18.851 to 25.860			18.781	1.886	15.072 to 22.490			8.492	1.514	5.515 to 11.470
Between one and two hours per day*Time2			20.732	1.576	17.632 to 23.833			16.697	1.557	13.634 to 19.760			9.303	1.453	6.445 to 12.161
Between two and five hours*Time1			19.478	1.214	17.089 to 21.866			16.379	1.313	13.798 to 18.961			7.957	1.114	5.766 to 10.148
Between two and five hours*Time2			20.870	1.070	18.765 to 22.974			17.014	1.017	15.014 to 19.014			8.575	0.964	6.678 to 10.472
More than five hours*Time1			21.264	1.743	17.836 to 24.693			18.141	1.539	15.115 to 21.168			10.278	0.964	7.365 to 13.191
More than five hours*Time2			19.830	1.485	16.909 to 22.751			17.194	1.340	14.558 to 19.829			11.188	1.511	8.216 to 14.159
How often do you search for information about the progress of the epidemic?*Time	0.755	0.471				0.106	0.899				1.082	0.340			
Rarely *Time1			16.797	1.490	13.867 to 19.727			15.164	1.554	12.107 to 18.221			8.240	1.310	5.664 to 10.815
Rarely *Time2			16.891	1.313	14.308 to 19.475			14.759	1.228	12.343 to 17.175			6.561	1.215	4.172 to 8.950
Once a day*Time1			19.202	1.575	16.104 to 22.300			17.832	1.506	14.871 to 20.794			8.039	1.406	5.272 to 10.805
Once a day*Time2			21.529	1.352	18.871 to 24.188			17.984	1.251	15.524 to 20.444			8.537	1.212	6.152 to 10.922
Many times a day*Time1			21.929	1.589	18.803 to 25.054			17.107	1.621	13.919 to 20.296			9.099	1.488	6.173 to 12.025
Many times a day*Time2			21.682	1.284	19.157 to 24.207			16.226	1.152	13.960 to 18.491			10.106	1.157	7.830 to 12.381
MAAS	8.806	0.003 **				6.942	0.009**				2.075	0.151			
Brief-COPE Approach	0.188	0.665				5.166	0.024*				0.292	0.590			
Brief-COPE Avoidant	56.920	<0.001**				76.244	<0.001***				25.116	<0.001***			

Results refer to the three regression linear models with DASS-21 Anxiety, DASS-21 Stress and DASS-21 Depression as dependent variables, linked to the ANCOVA model. Covariates in the model are MAAS, Brief-COPE Avoidant and Brief-COPE Approach. Covariates are evaluated based on the following values: MAAS = 57, Brief-COPE Avoidant = 27, Brief-COPE Approach = 36. *T*-tests are evaluated at 5% (*p < 0.05), 1% (**p < 0.01), and 0.1% (***p > 0.001).

The overarching goal of the survey was to investigate COVID-19 awareness, coping strategies, the psychological implications as well as the role of technology as a vital source of communication and information during lockdowns (Luo et al., 2020).

To measure these aspects, self-administered psycho-diagnostic tests were used. These tests were previously validated in the international and Italian research (Moccia et al., 2020; Wang C. et al., 2020; Wang H. et al., 2020; Passavanti et al., 2021) and served the dual purpose of assessing individual characteristics and identifying the presence of psychopathologies.

This study utilized a range of established psychometric tools to assess various psychological aspects influenced by the COVID-19 pandemic.

The **Mindfulness Awareness Attention Scale (MAAS),** a 15-item questionnaire, evaluated attention and mindfulness, recognized for its reliability and strong associations with meditation and self-awareness, where higher scores indicate increased mindfulness (Brown and Ryan, 2003).

The **Impact or Event Scale-Revised (IES-R),** comprising 22 items on a 5-point Likert scale, measured PTSD, encompassing sub-dimensions of intrusiveness, hyper-arousal, and avoidance. A total score of 33 suggested potential PTSD, with the option to categorize psychological impact as normal, mild, moderate or severe (Creamer et al., 2003; Wang C. et al., 2020; Wang H. et al., 2020).

The **Depression, Anxiety, and Stress Scale (DASS-21)** assessed psychological constructs such as depression, anxiety, and stress using a 21-item self-report scale on a 4-point Likert scale. While it did not provide clinical diagnoses, it gauged severity, with scores multiplied by 2 to indicate levels from normal to extremely severe (Henry and Crawford, 2005).

The **Patient Health Questionnaire (PHQ-9)** employed a brief 4-point Likert scale to screen for mental health conditions, primarily depression, and considered functional impairment in daily activities. Scores ranged from 0 to 27, reflecting varying degrees of depression (Kroenke et al., 2001).

The **Perceived Stress Scale (PSS10)** was used to ascertain perceived stress, utilizing a 5-point Likert scale ranging from 0 to 4. The total score categorized stress levels as low, moderate, or high (Cohen et al., 1983).

The **Brief-COPE**, a concise version of the COPE, identified common coping strategies, categorizing them into avoidance (denial, substance use, venting, behavioral disengagement, self-distraction, guilt) and approach (active coping, positive reframing, planning, acceptance, seeking emotional support, seeking informational support) (Carver, 1997; Meyer, 2001).

These assessments were validated versions drawn from prior international research (Chew et al., 2020; Conversano et al., 2020; Dawson and Golijani-Moghaddam, 2020; Tee et al., 2020; Umucu and Lee, 2020; Yao, 2020; Yan et al., 2021). This approach enhances the study’s applicability and offers a comprehensive understanding of the psychological repercussions of the COVID-19 pandemic across diverse cultural contexts, Italy included.

### 2.3 Statistical analysis

The data were analysed using the software package IBM SPSS Statistics version 26. The method applied was the analysis of covariance (ANCOVA), which combines the analysis of variance (ANOVA) and linear regression covariates. The psychological effects of the pandemic (anxiety, stress, depression, and post-traumatic stress disorder) during spring 2020 and spring 2021 were compared using a generalized linear model for repeated measurement. The model assumptions were found to be fulfilled with correlation between dependent and covariate variables and non-correlation between independent and covariate (Tables 1, 2).

In this research, the statistical analysis involved a sample of 184 Italian respondents, identifying the results related to the main psychological constructs considered.

The analysis was performed six times to elaborate the averages of the dependent variables related to the onset of psychopathological symptoms. Considering the previously cited existing literature and the follow-up nature of this research, variables related to coping strategies (Brief-COPE) and mindfulness (MAAS) were identified as covariates.

## 3 Results

### 3.1 Sociodemographic characteristics

In 2021, this study examines the psychological impact of COVID-19. Unlike the previous survey, which included participants from seven countries (Australia, China, Ecuador, Iran, Italy, Norway and the United States), this follow-up specifically targets Italian individuals.

The sample comprises 184 participants, consisting of 56 males (30.4%) and 128 females (69.6%). The average age is 27.22 (SD = 7.60). In a preliminary analysis, age did not appear to be a determining factor for stress, depression, anxiety, and post-traumatic stress disorder (PTSD). Among the interviewees, 173 (94%) do not have children, while 11 (6%) have one child or more.

The survey also collects data on cultural and economic factors, including family income, occupation (study, work, or neither) and education. Within the interviewed sample, 35.3% of respondents (65) identify themselves as low-income, 47.3% (87) as medium-income, and 17.4% (32) as high-income. In terms of occupation, 7.1% are unemployed, 55.4% are students, 24.4% are workers, and 2.2% are both students and workers. Regarding education, 53 interviewees (28.7%) have a middle school education, 68 (37%) hold a bachelor’s degree and 63 (34.3%) have at least a master’s degree.

### 3.2 Psychological impact

**Figure 1:**
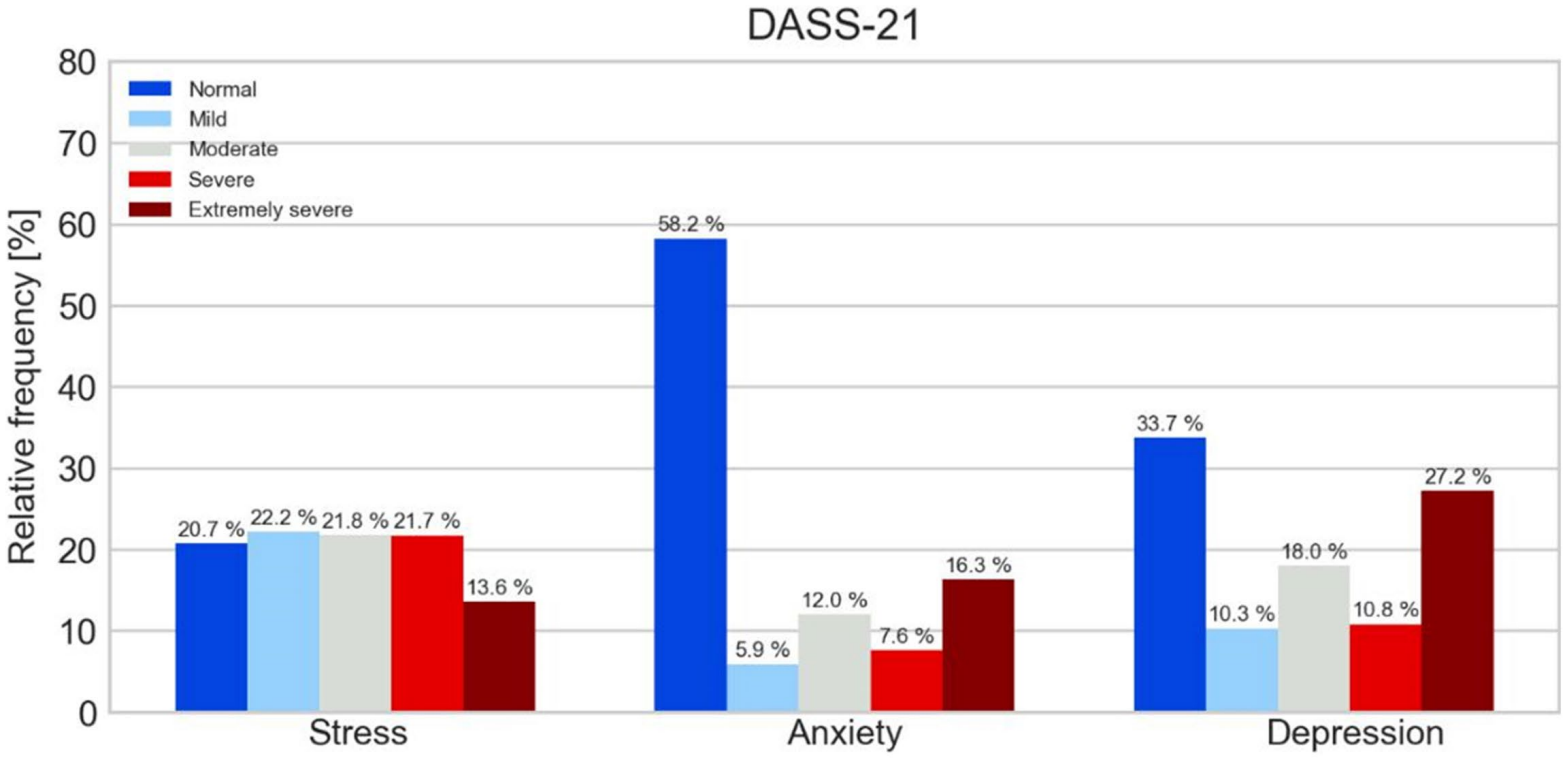
Relative frequencies of DASS-21 subscales in the sample.

In Figure 1, the DASS-21 Stress subscale indicates 76 respondents (20.7%) with normal scores, 41 (22.2%) with mild stress, 40 (21.8%) with moderate stress, 40 (21.7%) with severe stress and 25 (13.6%) with extremely severe stress.

For the DASS-21 Anxiety subscale, 107 participants (58.2%) have normal scores, 11 (5.9%) mild anxiety, 22 (12%) moderate anxiety, 14 (7.6%) severe anxiety and 30 (16.3%) extremely severe anxiety.

Regarding the DASS-21 Depression subscale, 62 respondents (33.7%) are related to normal scores, 19 (10.3%) mild depression, 33 (18%) moderate, 20 (10.8%) severe and 50 (27.2%) extremely severe.

**Figure 2:**
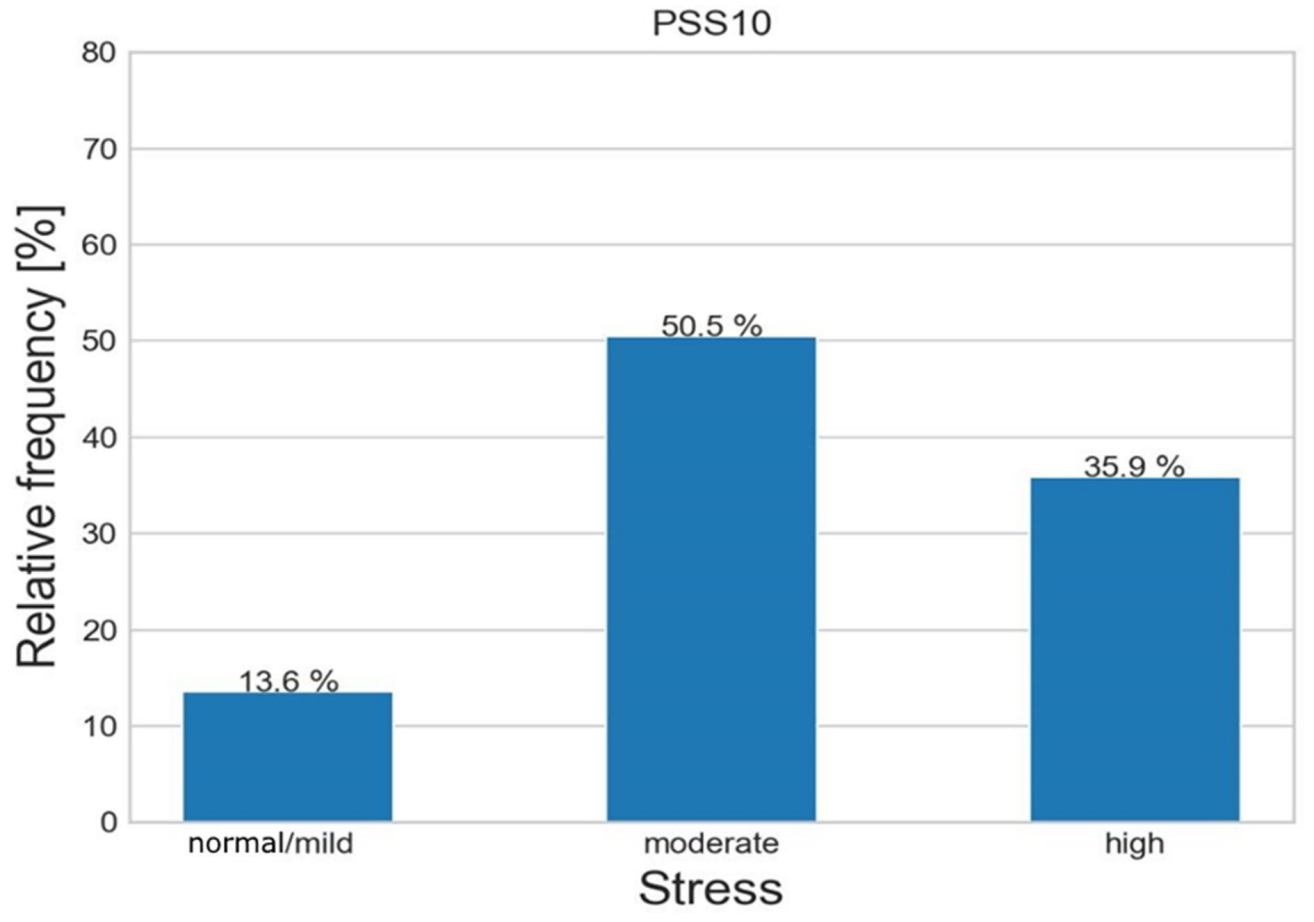
Relative frequencies of PSS10 scale in the sample.

In Figure 2, PSS10 results show mild or absent stress in 25 individuals (13.6%), moderate in 93 (50.5%) and high in 66 (35.9%).

**Figure 3:**
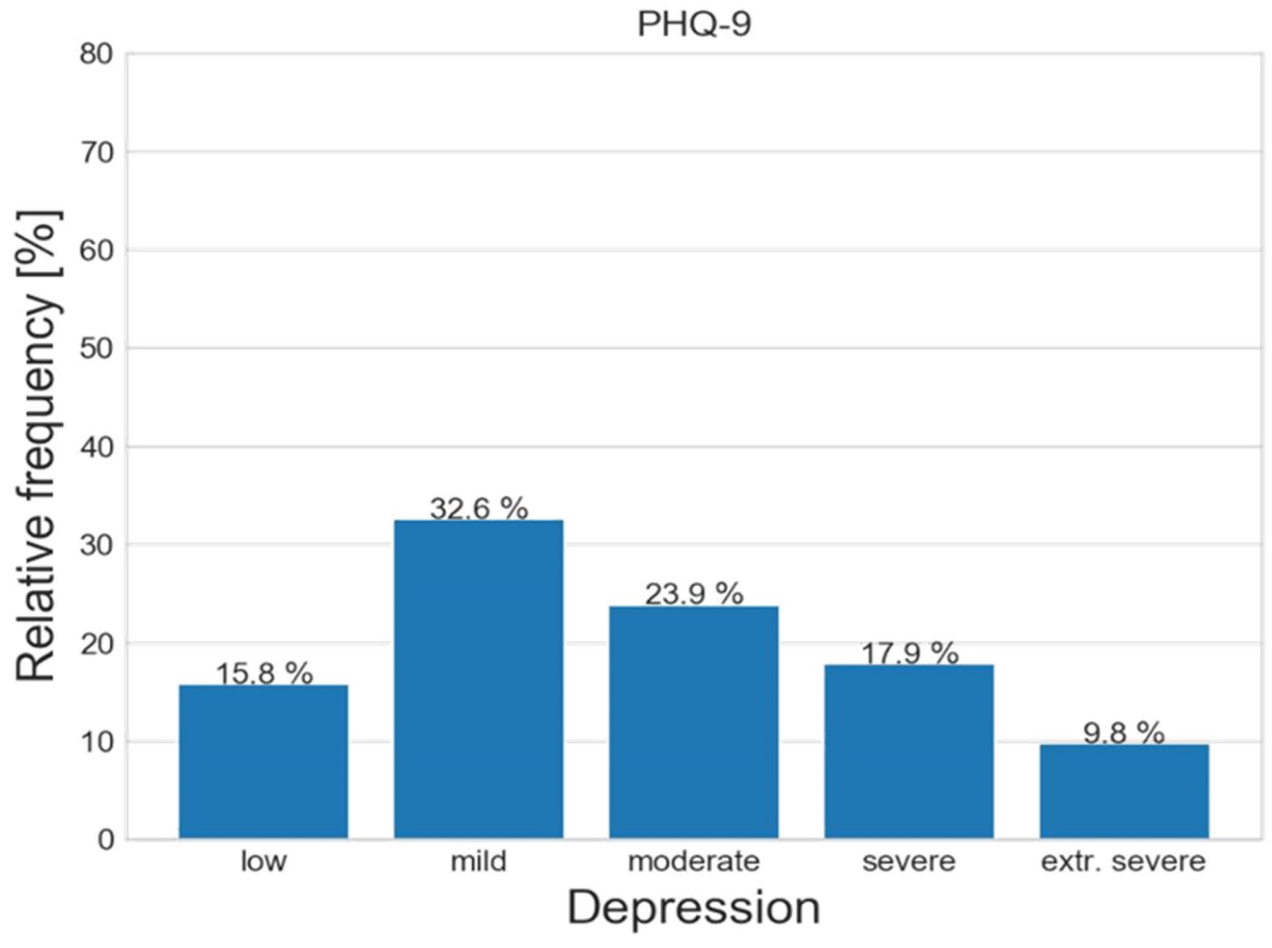
Relative frequencies of PHQ-9 scale in the sample.

Figure 3 displays PHQ-9 scores: 29 respondents (15.8%) do not display depression, 60 (32.6%) mild depression, 44 (23.9%) moderate, 33 (17.9%) moderately severe and 18 (9.8%) severe.

**Figure 4:**
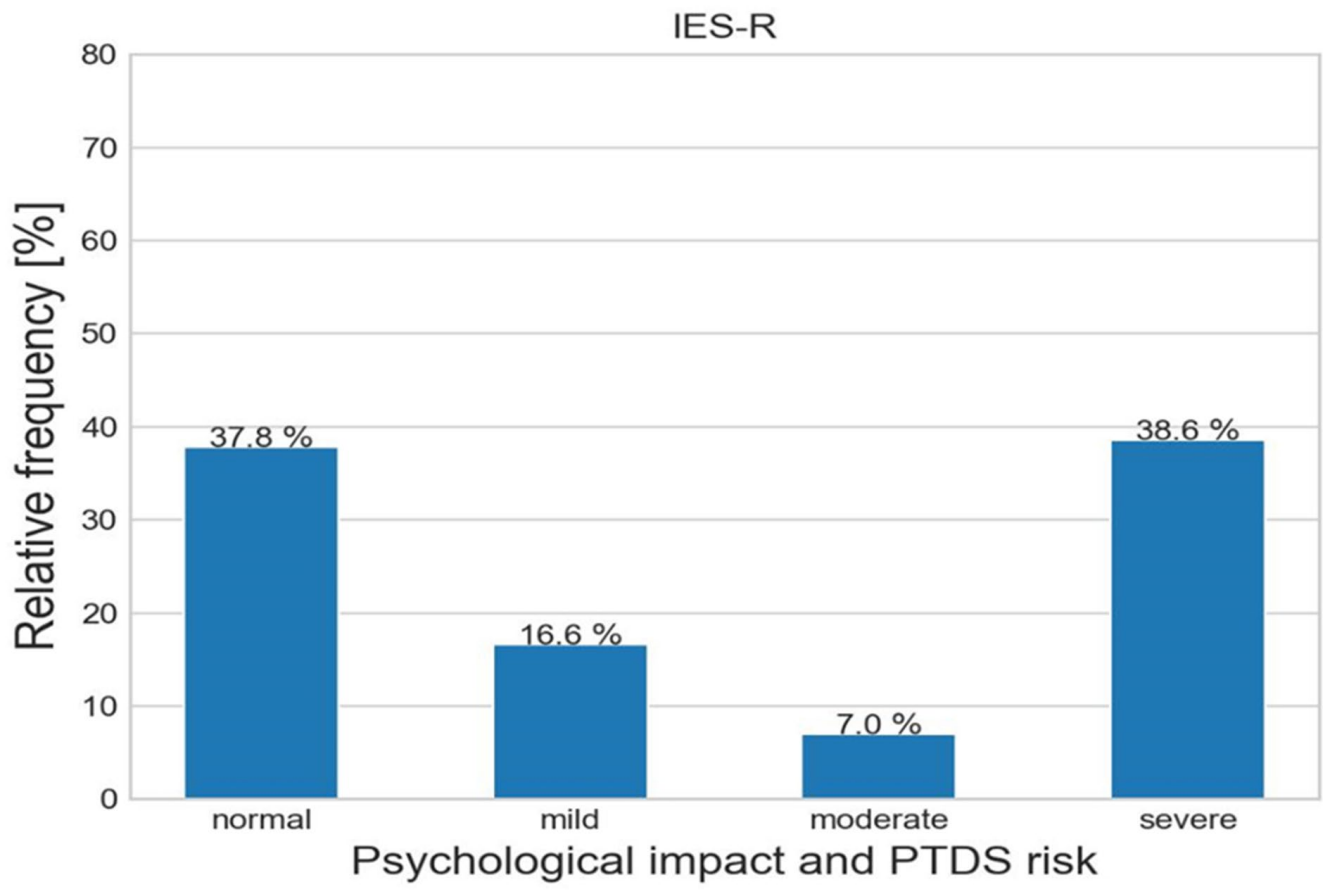
Relative frequencies of IES-R scale in the sample.

On the IES-R scale (Figure 4), 139 participants (37.8%) are characterized by normal scores, 31 (16.6%) mild psychological impact, 13 (7%) moderate psychological impact and 71 (45.5%) severe psychological impact.

On the MAAS scale, the average score is *M* = 56.68, SD = 13.82.

For the Brief-COPE Approach scale, the average value is *M* = 35.89, SD = 5.41, while on the Brief-COPE Avoidant scale the outcome is *M* = 26.56, SD = 4.51.

### 3.3 Model results

In line with the previous study, gender significantly affects scores across all six tests (Table 1). The PHQ-9 shows a significant gender difference (*F* (1,341) = 13.434, *p* < 0.001), with females scoring higher (*M* = 10.896, SE = 0.388) than males (*M* = 8.674, SE = 0.533).

Similarly, the IES-R test reveals gender disparities (*F* (1,341) = 7.002, *p* = 0.009), with females scoring higher (*M* = 34.537, SE = 1.480) than males (*M* = 28.561, SE = 1.952). In the PSS10, females (*M* = 23.258, SE = 0.546) outscore males (*M* = 19.815, SE = 0.730) significantly (*F* (1,341) = 16.017, *p* < 0.001).

On the DASS-21 subscales (Table 2), females exhibit higher average scores for Stress (*M* = 22.323, SE = 0.822), Anxiety (*M* = 10.046, SE = 0.833), and Depression (*M* = 18.301, SE = 0.906), with significant differences in groups means (MD = −5.302, *p* < 0.001; MD = −3.232, *p* = 0.005; MD = −3.577, *p* = 0.005), respectively.

Education does not significantly affect IES-R, PSS10, Stress, Anxiety, or Depression DASS-21 scales. However, in the PHQ-9, individuals with a master’s degree or Ph.D. (*M* = 8.769, SE = 0.512) score lower than those with a bachelor’s degree (*M* = 10.823, SE = 0.515), showing a significant difference (*F* (2,341) = 4.579, *p* = 0.011, MD = 2.055, *p* = 0.008).

Knowing someone who died from COVID-19 reveals significant differences in PHQ-9 (*F* (3,341) = 4.676, *p* = 0.031) and PSS10 (*F* (1,341) = 4.712, *p* = 0.031) scores, with lower scores among those acquainted with COVID-19-related deaths. Similarly, on the DASS-21 Depression subscale, a notable difference (*F* (1,341) = 6.996, *p* = 0.009; MD = 2.952, *p* = 0.009) is observed between those acquainted with COVID-19 deaths and those who are not, with lower scores for the former ones. No significant differences are found on the Stress, Anxiety DASS-21 subscales, or the IES-R subscale.

### 3.4 Use of means of information and communication

Regarding time spent on social networks, no significant differences were observed in the three DASS-21 subscales, IES-, and PSS10 scales. However, on the PHQ-9 scale, individuals spending less than one hour per day (*M* = 7.126, SE = 0.954) scored significantly lower than those spending one to two hours (MD = −3.359, *p* = 0.005), two to five hours (MD = −3.585, *p* = 0.005), and over five hours (MD = −3.693, *p* = 0.005).

Concerning the time spent on gathering pandemic-related information, a significant difference emerges on the IES-R scale (*F* (2,341) = 9.623, *p* < 0.001) and DASS-21 Stress subscale (*F* (2,341) = 5.654, *p* = 0.004). Survey participants who spent less time searching for information scored lower on the IES-R scale (*M* = 25.707, SE = 1.926) compared to those with moderate (MD = −6.938, *p* = 0.008) and high (MD = −10.588, *p* < 0.001) information research frequency. Similarly, on the DASS-21 Stress subscale, respondents with a low information-seeking frequency display a lower score (*M* = 16.844, SE = 1.132) than those with moderate (MD = −3.522, *p* = 0.026) and high frequency (MD = −4.961, *p* = 0.006).

### 3.5 Awareness and coping strategies

Covariances in the model link coping mindful awareness strategies to variations in dependent variables. Low MAAS scores, indicating reduced awareness, significantly associate with high values of PHQ-9, IES-R, PSS10, and DASS-21 Stress and Depression subscales. Further, higher avoidance strategy attitudes (Brief-COPE Avoidant) are related to elevated scores on all scales. Eventually, higher Approach Strategy scores (Brief-COPE Approach) correspond to significantly lower PSS10 and DASS-21 Depression subscale values. These results are reported in Tables 1, 2.

### 3.6 Comparison of the psychological impact on the survey population in 2020 and 2021

This subsection compares the findings from the initial survey conducted in spring 2020 with those from the follow-up in 2021 using a repeated measures ANCOVA. No significant differences are found between the scores on the three DASS-21 subscales as well as in the PHQ-9 and PSS10 tests for both years.

**Figure 5:**
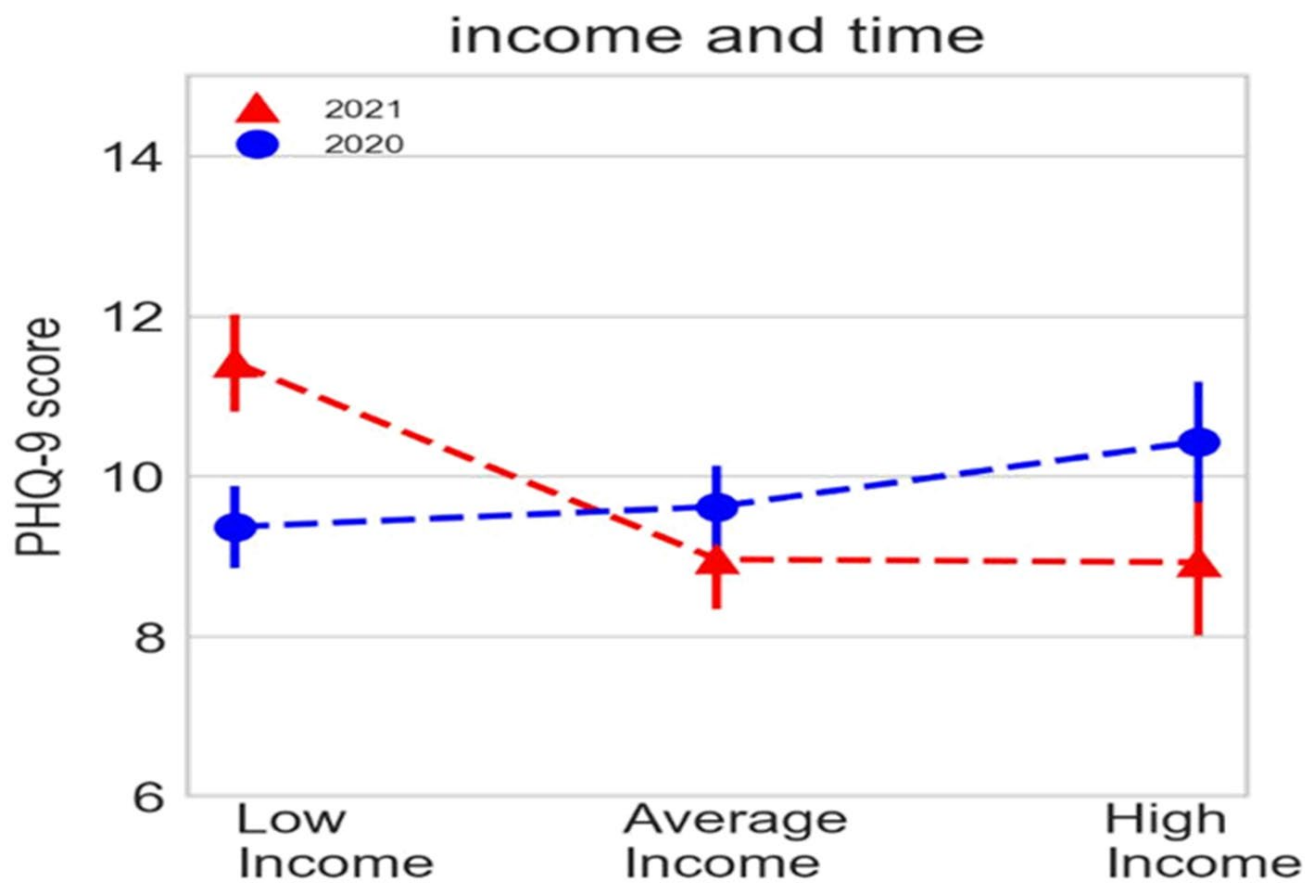
Relative frequencies of PHQ-9/Income and time scale in the sample.

However, a notable difference emerges on the IES-R scale (*F* (1,341) = 12.255, *p* = 0.001) as in 2021, participants scored lower (*M* = 28.836, SE = 1.292) compared to 2020 (*M* = 34.262, SE = 1.726). Furthermore, a temporal effect is observed in relation to other variables. For example, the PHQ-9 scores are influenced by income in both 2020 and 2021 (Figure 5). In 2020, scores are (*M* = 9.365, SE = 0.612) for low income, (*M* = 9.618, SE = 0.623) for medium income, and (*M* = 10.433, SE = 0.910) for high income, with no significant difference. However, in 2021, the low-income group shows an increase in scores (*M* = 11.414, SE = 0.511) indicating a significant difference *F* (2,341) = 6.139, *p* = 0.002 compared to the average (MD = −2.458, *p* = 0.001) and high-income (MD = −2.491, *p* = 0.010) groups, whose scores slightly decrease.

## 4 Discussion

While limited to Italian respondents, this study underscores the impact of COVID-19 and government containment measures on psychological well-being. The analysis of online questionnaires reveals heightened stress levels in 80 to 86% of the population, while depression rates range from 66 to 85% while anxiety affects around 42% of participants. Furthermore, 62% of the population is at risk of developing PTSD.

It is interesting to note that these results both tend to confirm and provide some additional indications compared to broader systematic reviews conducted in the meantime.

For instance, literature has highlighted an increase in anxiety and depression, particularly in the presence of pre-existing conditions or COVID-19 infections, as well as certain risk factors including female gender, being a nurse/healthcare worker, lower socio-economic status and social isolation. Meanwhile, some protective factors would include the presence of sufficient medical resources as well as up-to-date and accurate information (Luo et al., 2020; Khoodoruth et al., 2021).

Despite some recent long-term studies, it appears that the psychological impact of COVID-19 lockdowns is not as severe and uniform as previously thought, with significant yet relatively small effect sizes for anxiety and depression. Furthermore, some meta-regression analyses did not find significant moderation effects for average age, gender, continent, COVID-19 death rate or days of lockdown. This suggests that the restrictions do not affect everyone’s mental health in the same way, as many individuals seem to display psychological resilience to these impacts (Prati and Mancini, 2021). It appears necessary to delve further into this matter to increase the available data, as current findings sometimes seem contradictory.

Equally, these results align with prior research, highlighting the connection between awareness, coping strategies and psychological outcomes (Main et al., 2011; Passavanti et al., 2021; Smida et al., 2021). Lower scores on the Mindful Attention Awareness Scale (MAAS) correlate with increased stress, anxiety, depression and PTSD risk. Strategic coping approaches correspond to reduced PTSD risk and lower DASS-21 depression subscale scores, whereas avoidance strategies heighten psychopathological risk across various scales.

Gender differences exert a significant role, with females displaying significantly higher scores on subscales, indicating greater exposure to pandemic effects. These findings align with other studies (Li et al., 2020; Wang C. et al., 2020; Wang H. et al., 2020; Khoodoruth et al., 2021) highlighting that the female population is more susceptible to pandemic-related impacts.

Regarding education, a notable difference is observed on the PHQ-9, indicating that individuals with a bachelor’s degree experience higher stress levels than those holding a master’s degree or PhD.

Interestingly, respondents acquainted with someone who died due to COVID-19 exhibit lower scores on both the DASS-21 depression subscale and the PHQ-9 scale, possibly suggesting a degree of acceptance of the global situation. However, those with positive acquaintances displayed higher stress and anxiety levels.

The study also delved into the psychological effects of social media exposure and information retrieval frequency during the pandemic. The results show that individuals spending less than an hour per day on social networks have lower stress levels than those with greater exposure. The same consideration applies to those devoting less free time to pandemic-related information. These results corroborate existing literature emphasizing the psychological impact related to communication and information during crises (Neria and Sullivan, 2011; Roy et al., 2020; Shuja et al., 2020). In this regard, the intervention of institutions and media should aim to inform the general public in a fair and unbiased manner (Chand, 2021; Himelein-Wachowiak et al., 2021).

Throughout the pandemic period, no significant increase in stress, anxiety, or depression is observed among the participants over the eighteen-month timeframe following the outbreak in Italy. However, an elevated risk of PTSD is identified.

Regarding socioeconomic variables and exposure to social media and information, no significant changes are noted, except for a rise in stress levels among the low-income survey population during the second follow-up research. This outcome could be attributed to the Italian government countermeasures, resulting in income and job losses that disproportionately affected the low-income segment of the population. Despite this data being significant in line with what is found in the literature, it is equally important to emphasize how it may be biased due to self-categorization by individuals and their self-perception. For these reasons, it is important to consider it only within the broader context of the research and its limitations.

A worsening of the living standards, unemployment and the lack of career prospects have long been associated to higher stress levels. Similar results were found also in other countries, amongst them the UK (Shevlin et al., 2020) and the USA (Wolfson et al., 2021).

In general, many of the findings of this research echo a good portion of the considerations and outcomes valid for the previous study performed in 2020. In this regard, the time factor seems to exert a very limited impact on psychological distress, although specific results may be affected by the small sample size and various country-specific circumstances. Furthermore, it has been observed in the literature how organizational aspects related to lockdown policies and some precautionary measures, such as hand hygiene and mask-wearing, have an impact on psychopathological symptoms (Wang C. et al., 2020; Wang H. et al., 2020).

Finally, it is important to consider how some variables not considered in this research could be important mediators regarding the reported correlations, for example, the role that the presence of psychological support may have in such a timeframe. Similarly, it is important to note that a significant number of the initial 420 participants dropped out during the follow-up study in this phase, constituting an additional limiting factor that could partially impact the results.

## 5 Conclusion

This study has delved into the profound psychological distress induced by the COVID-19 pandemic and associated containment measures in Italy. Focusing on 184 individuals who participated in two online surveys (one in spring 2020 and a follow-up in winter 2021), this research has pivoted on a longitudinal analysis over eighteen months. The findings have revealed a significant prevalence of heightened stress, depression and anxiety levels, with around 62% of the population at risk of post-traumatic stress disorder (PTSD), particularly affecting women.

The study has highlighted the interplay between awareness levels, coping strategies and psychological impact. Lower scores on the Mindfulness Awareness Attention Scale (MAAS) correlate with increased stress, anxiety, depression and higher PTSD risk. Likewise, avoidance coping strategies worsen psychopathological risks.

Moreover, continuous exposure to traumatic media content and misinformation has negatively impacted mental well-being. Conversely, individuals who have limited their social media usage to less than an hour per day report lower stress levels.

An increased psychological risk has been observed among low-income participants during the second year of the pandemic. Economic repercussions, income loss and job displacement have contributed to this increment, mirroring similar findings in other countries. Therefore, the findings of this research hold significant implications for mental health interventions and policy development in the context of the COVID-19 pandemic.

Broadly speaking, this investigation has underscored the critical need for tailored mental health interventions aimed at addressing heightened levels of stress, anxiety, depression and the risk of developing PTSD among the population. The prevalence of these psychological challenges, particularly among certain demographic groups such as women and individuals with lower socioeconomic status, has highlighted the importance of targeted intervention strategies.

Furthermore, the findings emphasize the crucial role of coping mechanisms and mindful awareness in mitigating the psychological impact of the pandemic. Interventions that promote effective resilient strategies and mindfulness practices could serve as protective factors against adverse mental health outcomes.

In terms of policy development, the results indicate the importance of ensuring access to accurate and up-to-date information, as well as the responsible use of social media platforms. Regulations aimed at disseminating reliable news and combating misinformation could help alleviate unnecessary stress and anxiety among the population.

Moreover, the outcomes have highlighted the disproportionate impact of the pandemic on the most vulnerable groups of the population, such as those with lower income levels. Policy initiatives aimed at addressing socioeconomic disparities and providing support to marginalized communities are crucial for promoting mental well-being and resilience.

This study has underscored the urgent need for comprehensive interventions and evidence-based policy measures to address the psychological toll of COVID-19. By prioritizing mental health support and implementing targeted policies, it is possible to effectively mitigate the adverse effects of the pandemic and promote resilience within our societal communities.

## Data availability statement

The raw data supporting the conclusions of this article will be made available by the authors, without undue reservation.

## Ethics statement

The studies involving humans were approved by Norwegian Centre for Research Data. The studies were conducted in accordance with the local legislation and institutional requirements. The participants provided their written informed consent to participate in this study.

## Author contributions

IR: Conceptualization, Project administration, Supervision, Visualization, Writing – original draft, Writing – review & editing. ML: Formal analysis, Writing – original draft. MM: Resources, Writing – original draft. AA: Data curation, Formal analysis, Methodology, Writing – review & editing. AB: Investigation, Writing – review & editing. BL: Conceptualization, Project administration, Writing – review & editing. DB: Data curation, Methodology, Project administration, Writing – review & editing. MP: Conceptualization, Data curation, Formal analysis, Investigation, Methodology, Project administration, Resources, Supervision, Validation, Visualization, Writing – original draft, Writing – review & editing.
